# Molecular basis for protection and cross-protection by human antibodies targeting the parainfluenza virus hemagglutinin-neuraminidase protein

**DOI:** 10.1128/jvi.00502-26

**Published:** 2026-07-31

**Authors:** Katelyn D. McCaffrey, Behrouz Ghazi Esfahani, Mohamed A. Elbehairy, Anna L. McCormick, Jarrod J. Mousa

**Affiliations:** 1 Department of Biomedical Sciences, College of Medicine, Florida State University 7823, Tallahassee, Florida, USA; 2 Department of Poultry Diseases, Faculty of Veterinary Medicine, Cairo University 63526, Giza, Egypt; 3 Department of Infectious Diseases, College of Veterinary Medicine, University of Georgia 1355, Athens, Georgia, USA; Emory University School of Medicine, Atlanta, Georgia, USA

**Keywords:** cryo-EM, monoclonal antibodies, parainfluenza viruses

## Abstract

Human parainfluenza viruses (PIVs) are a leading cause of respiratory illness, particularly in vulnerable populations, where infection can lead to severe disease. Despite their clinical impact, there are currently no licensed vaccines or effective antiviral treatments available. PIVs have two large surface proteins, the fusion and hemagglutinin-neuraminidase (HN) proteins, both of which are targets of neutralizing antibodies. In this study, we identified and characterized two human monoclonal antibodies (mAbs), 5217-2 and 5217-9, which bind recombinant PIV3 HN protein, recognize PIV3-infected cells, and neutralize *in vitro*. We determined the antigenic sites of the PIV3 HN-specific mAbs via biolayer interferometry and found mAb 5217-9 targets a previously defined neutralizing antigenic site, while mAb 5217-2 binds a distinct antigenic site, expanding the known antigenic landscape. To further understand the newly defined epitope, we determined a cryo-electron microscopy (cryo-EM) structure of mAb 5217-2 in complex with PIV3 HN, revealing an epitope adjacent to the PIV3 HN protein receptor binding site. We also determined the structure of the previously discovered anti-PIV3 HN mAb PIV3HN-09, previously shown to be partially protective *in vivo*. In a hamster challenge model of PIV3, mAb 5217-2 significantly reduced lung viral titers, demonstrating its protective capacity. Furthermore, as the mAb PIV3HN-05 was previously shown to cross-neutralize PIV1, we evaluated its protective efficacy in a PIV1 challenge model, demonstrating reduced lung viral titers. Overall, these findings provide new insights into PIV3 HN epitopes to support structure-based vaccine design and demonstrate protective mAbs for both PIV3 and PIV1.

**IMPORTANCE:**

Human parainfluenza viruses (PIVs) are a major cause of respiratory illness, yet there are currently no licensed vaccines or targeted antiviral therapies. A better understanding of how antibodies recognize viral surface proteins is critical for developing effective interventions. In this study, we identified human monoclonal antibodies that neutralize PIV3 and mapped the specific regions of the viral hemagglutinin-neuraminidase (HN) protein they recognize. Structural analysis revealed a previously uncharacterized antibody-binding site near the HN active site and expanded the known antigenic landscape of this protein. We also demonstrate protective activity of these antibodies in animal models against PIV3 and PIV1 infection. These findings provide new targets and structural insights that can guide the development of vaccines and antibody-based therapies against PIVs.

## INTRODUCTION

Human parainfluenza viruses (PIVs) are a leading cause of acute respiratory infection (ARI) in children, immunocompromised individuals, and the elderly (1). PIVs can cause both upper and lower respiratory tract infections, typically presenting with cold-like symptoms, or in more severe cases can result in croup, bronchitis, pneumonia, which can lead to hospitalization (1, 2). PIV infections are often underrecognized due to the annual epidemics of respiratory syncytial virus (RSV) and influenza virus; however, PIVs remain a major cause of ARIs in children and are consistently among the leading viral contributors to pediatric respiratory disease (3). PIVs tend to severely affect young children, with those under five years of age exhibiting the highest PIV-associated hospitalization rates (3–5). Among these cases, a substantial proportion occurs in infants, particularly those under one year of age, contributing significantly to infant morbidity and, less commonly, severe disease and mortality (4, 6). In elderly and immunocompromised populations, PIV infection frequently progresses from upper to lower respiratory tract disease and is associated with high rates of pneumonia, prolonged viral shedding, and increased mortality (1, 7, 8). These individuals are also particularly susceptible to nosocomial transmission and outbreaks in nursing homes (1, 8).

There are five known serotypes of parainfluenza viruses that infect humans: PIV1, PIV2, PIV3, PIV4a, and PIV4b (9). These serotypes differ in their epidemiology and clinical severity. Among them, PIV3 has been associated with lower respiratory tract illness, with symptoms including bronchiolitis and pneumonia, and has been consistently linked to the highest hospitalization rates, reflecting its potential for causing severe respiratory disease (10–12). PIV1 follows closely behind and is a major contributor to hospital admissions in children under 1 year of age, most frequently presenting as croup and cold-like symptoms, but it can progress to more severe disease requiring hospitalization (10, 12). In comparison, PIV2 is generally associated with upper respiratory illness, with common cold-like symptoms and lower rates of hospitalization (11, 13). PIV4a and PIV4b remain understudied due to limited historical testing; however, recent studies suggest that these viruses cause respiratory disease comparable to PIV3, with infection of the lower respiratory tract and are associated with severe outcomes (4, 14).

The PIVs are negative-sense, single-stranded RNA viruses belonging to the *Paramyxoviridae* family. PIV1 and PIV3 are classified under the genus *Respirovirus*, while PIV2 and PIV4 fall within the genus *Orthorubulaviru*s, a taxonomic revision of the former genus *Rubulavirus* (13, 15, 16). The RNA genome of PIVs encodes at least six viral proteins: nucleocapsid protein (NP), phosphoprotein (P), matrix protein (M), fusion glycoprotein (F), hemagglutinin-neuraminidase glycoprotein (HN), and RNA polymerase (L) (17, 18). The PIV HN and F surface glycoproteins play critical roles in initiating infection. The PIV HN protein is a type II integral membrane protein composed of a stalk region and a globular head domain, which can assemble as a homodimer or homotetramer (19). Unlike many other receptor-binding glycoproteins in the Paramyxovirus family, PIV HN possesses dual functionality: it uses hemagglutinin activity to bind sialic acid on host cells and neuraminidase activity to cleave sialic acid and release viral particles during budding (18, 20–22). In addition to mediating attachment to sialic acid-containing receptors, the PIV HN protein plays a critical role in activating the F protein (21, 23). The PIV F protein mediates the fusion of the viral envelope with the host cell membrane, enabling viral entry (21, 23). Upon activation, the PIV F protein undergoes a conformational change that exposes its buried fusion peptide, enabling the fusion of the viral and host cell membranes (23, 24). Both the PIV HN and F proteins are major targets for neutralizing antibodies, making them key candidates for therapeutic and vaccine development (22). The virus relies heavily on the PIV HN protein functions, including receptor binding, receptor cleavage, and PIV F protein activation, making it a strategic target for interventions aimed at disrupting receptor binding, thus preventing membrane fusion. While the PIV F protein has been the focus of many antiviral strategies, the multifunctional nature of the PIV HN protein highlights its potential as a more advantageous therapeutic target (22, 25, 26).

Currently, there are no approved vaccines for PIVs, despite decades of research efforts. Multiple vaccine platforms have been evaluated in both preclinical and clinical trials, including live-attenuated, vector-based, subunit, and mRNA vaccine candidates (27). Among the most promising candidates, rPIV3cp45, a recombinant, live-attenuated PIV3 vaccine, has shown safety in infants and young children and induces an immune response against the virus (27–30). Other serotypes have also been clinically tested: a live-attenuated PIV1 vaccine candidate, rPIV-1/84/del170/942A, was safe in adults and children but was over-attenuated, resulting in lower immune responses in PIV1-seronegative children (31). Similar findings were reported for the live-attenuated PIV2 vaccine candidates (32). More recently, mRNA vaccine platforms have been explored due to the success of the COVID-19 mRNA vaccines. An mRNA-based candidate targeting the F glycoprotein on both PIV3 and human metapneumovirus (hMPV) was shown to induce immune responses against both viruses in adults and seropositive children (33, 34).

Increasing attention is being directed toward monoclonal antibodies (mAbs) as a potential preventative and therapeutic strategy. mAbs are highly specific proteins that can target conserved viral or bacterial antigens and have been reported with favorable safety profiles across numerous clinical studies (35–37). In the case of PIVs, where immunocompromised individuals are at a heightened risk for severe disease, passive immunization with mAbs offers a safe approach for providing immediate, antigen-specific protection (38). Significant advances have been made in mAb research for respiratory viruses, such as RSV, which has three commercialized mAbs to reduce disease burden in infants (27): palivizumab, nirsevimab, and clesrovimab all target the RSV fusion protein and have been approved to provide passive immunity in high-risk and/or healthy infants (39–41). In addition, several mAbs targeting PIVs have demonstrated protective efficacy in animal models (27). PIA174, a mAb targeting the PIV3 F protein, has been shown to reduce viral load in both the lungs and nasal passages of cotton rats (42, 43). Furthermore, the broadly reactive mAb 3x1 targets the F proteins of both PIV3 and PIV1, and when administered as a cocktail with the mAb MxR (which neutralizes RSV and hMPV), has been shown to significantly reduce lung viral titers against all four viruses (44). The success of mAb-based therapies offers a promising strategy for preventing and treating PIV infections, particularly in populations at high risk of severe disease.

The absence of effective treatments allows human parainfluenza viruses to remain a significant threat to vulnerable populations. In this paper, we identified two new neutralizing human mAbs, 5217-2 and 5217-9, targeting the HN protein of PIV3. Each of these mAbs was found to target a distinct epitope on the PIV3 HN protein, and we were able to characterize the structural features of these epitopes via epitope binning and cryo-electron microscopy (cryo-EM). Prophylactic administration of mAb 5217-2 significantly reduced PIV3 replication *in vivo*. We also determined the cryo-EM structure of the previously discovered protective mAb PIV3HN-09 in complex with the PIV3HN protein. Finally, we determined that the previously discovered PIV1/PIV3 cross-neutralizing mAb PIV3HN-05 protects against PIV1 *in vivo*.

## RESULTS

### Binding and neutralizing properties of PIV3HN-targeting mAbs

**Fig 1:**
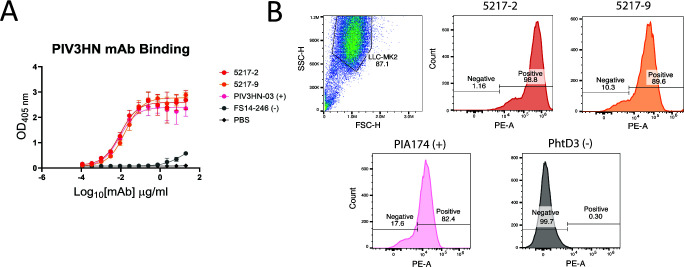
ELISA binding and mAb binding to virally infected cells. (**A**) ELISA binding curves of isolated mAbs, 5217-2 and 5217-9, against the PIV3 HN protein. Optical density at 405 nm (OD_405_) was measured using a microplate reader. Data points are the average of four technical replicates from one experiment and are representative of two biological replicates. The error bars represent the standard deviation. (**B**) LLC-MK2 cells were infected with PIV3 and stained with PE-conjugated PIV3 HN-specific mAbs 5217-2 and 5217-9. The anti-F mAb PIA174 was used as a positive control, and a mAb against *Streptococcus pneumoniae*, PhtD3, was used as a negative control. Both the HN-specific mAbs and anti-F mAb showed binding to PIV3-infected cells. The data represent one technical replicate from one experiment, which is representative of two biological replicates.

Peripheral blood mononuclear cells (PBMCs) were isolated from generally healthy human subjects and sorted for PIV3 HN-targeting B cells. Single-cell RNA sequencing of these B cells was performed using the 10× Genomics Chromium platform, followed by next-generation sequencing to recover paired heavy and light chain mAb sequences (45–47). We identified and recombinantly produced two mAbs that were specific to the PIV3 HN protein, termed 5217-2 and 5217-9. The binding of mAbs 5217-2 and 5217-9 to the PIV3 HN protein was confirmed via enzyme-linked immunosorbent assay (ELISA). Both mAbs demonstrated binding to the PIV3 HN protein, with mAb 5217-2 exhibiting an EC_50_ of 9 ng/mL and mAb 5217-9 exhibiting an EC_50_ of 17 ng/mL. A previously discovered PIV3 HN-binding mAb, PIV3HN-03, was used as a positive control and displayed a comparable EC_50_ of 9 ng/mL (Fig. 1A) (47). To confirm that our mAbs recognize the native full-length PIV3 HN protein, we evaluated their binding to PIV3-infected LLC-MK2 cells via flow cytometry. This technique allows the detection of viral proteins as they appear on the surface of infected cells, providing insights into whether the epitopes remain accessible during infection. Both mAbs 5217-2 and 5217-9 showed staining of PIV3-infected cells, comparable to the positive control anti-PIV3 F mAb PIA174. An absence of staining was observed by the negative control anti-pneumococcal mAb PhtD3 (48). Uninfected cells showed no detectable staining with either mAb, confirming the specificity of the observed signal for the PIV3-infected cells (Fig. 1B and Fig. S1). These data indicate that the epitopes recognized by mAbs 5217-2 and 5217-9 are preserved in the native structure of the PIV3 HN protein and are exposed during infection, supporting the functional relevance of the epitopes targeted by mAbs 5217-2 and 5217-9.

**Fig 2:**
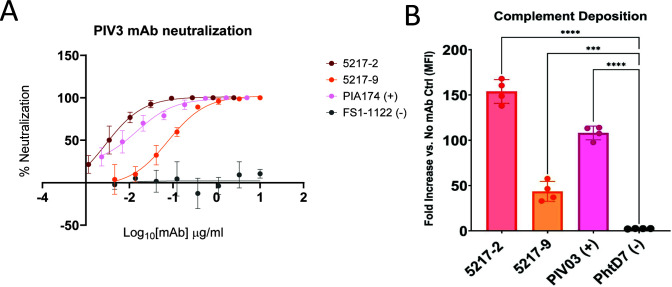
Virus neutralization of isolated mAbs and associated complement-dependent cytotoxicity assay. (**A**) Plaque reduction neutralization assay of the two isolated mAbs against PIV3. Data points are the average of three technical replicates from one experiment and are representative of two biological replicates. The data are presented using mean values ± standard deviation. (**B**) NeutrAvidin beads were coated with biotinylated PIV3 HN protein and incubated with individual mAbs. Guinea pig complement C3 was then added to the immune complexes. Complement deposition was analyzed using the Cytek Aurora flow cytometer and measured relative to C3 deposition in the absence of antibody. Data points represent the average MFI from two independent biological replicates, each with two technical replicates. The error bars represent the standard deviation. Statistical significance for the indicated comparisons versus PhtD7 (−) was determined using one-way ANOVA. ****P < 0.0001, ***P = 0.0001.

The mAbs were subsequently tested for their ability to neutralize PIV3 *in vitro* using a plaque reduction neutralization assay. This approach quantifies how effectively a mAb can inhibit viral infection by tracking the number of plaques, which are areas of cell death, formed in a cell monolayer. The degree of plaque reduction reflects the neutralizing strength of the mAb. mAb 5217-2 exhibited an IC_50_ of 3 ng/mL against PIV3, while mAb 5217-9 had an IC_50_ of 70 ng/mL, both of which are compared to the previously discovered anti-PIV3 F mAb PIA174 that had an IC_50_ of 15 ng/mL (Fig. 2A) (43).

### Complement deposition mediated by PIV3 HN-targeting mAbs

Beyond their ability to recognize and neutralize pathogens through their Fab regions, antibodies also play a crucial role in activating innate immune defenses. Through their Fc region, antibodies can interact with complement proteins, initiating a cascade that leads to the formation of the membrane attack complex and ultimately cell lysis of the target cell (49, 50). It was previously shown that anti-PIV3 HN mAbs can initiate this pathway, and mAbs targeting the previously described antigenic site 2 demonstrated the highest complement deposition activity (47). To determine whether the newly generated mAbs could initiate this pathway, we evaluated their capacity to promote C3 deposition when introduced to the PIV3 HN antigen. When complement is recruited to the antigen-antibody complex, C3 becomes deposited on the surface and can be detected using a FITC-conjugated anti-C3 antibody. Among the mAbs tested, 5217-2 induced the highest level of complement deposition, resulting in a 150-fold increase in C3 signal relative to the no mAb control. This level of activity exceeded that of the previously described PIV3HN-03, which had shown the strongest complement deposition within its original panel (47). In contrast, mAb 5217-9 elicited a weaker response of a 40-fold increase. As expected, the anti-pneumococcal mAb PhtD7 did not promote C3 deposition (Fig. 2B) (48).

### Epitope mapping reveals a novel antigenic site on the PIV3 HN protein

**Fig 3:**
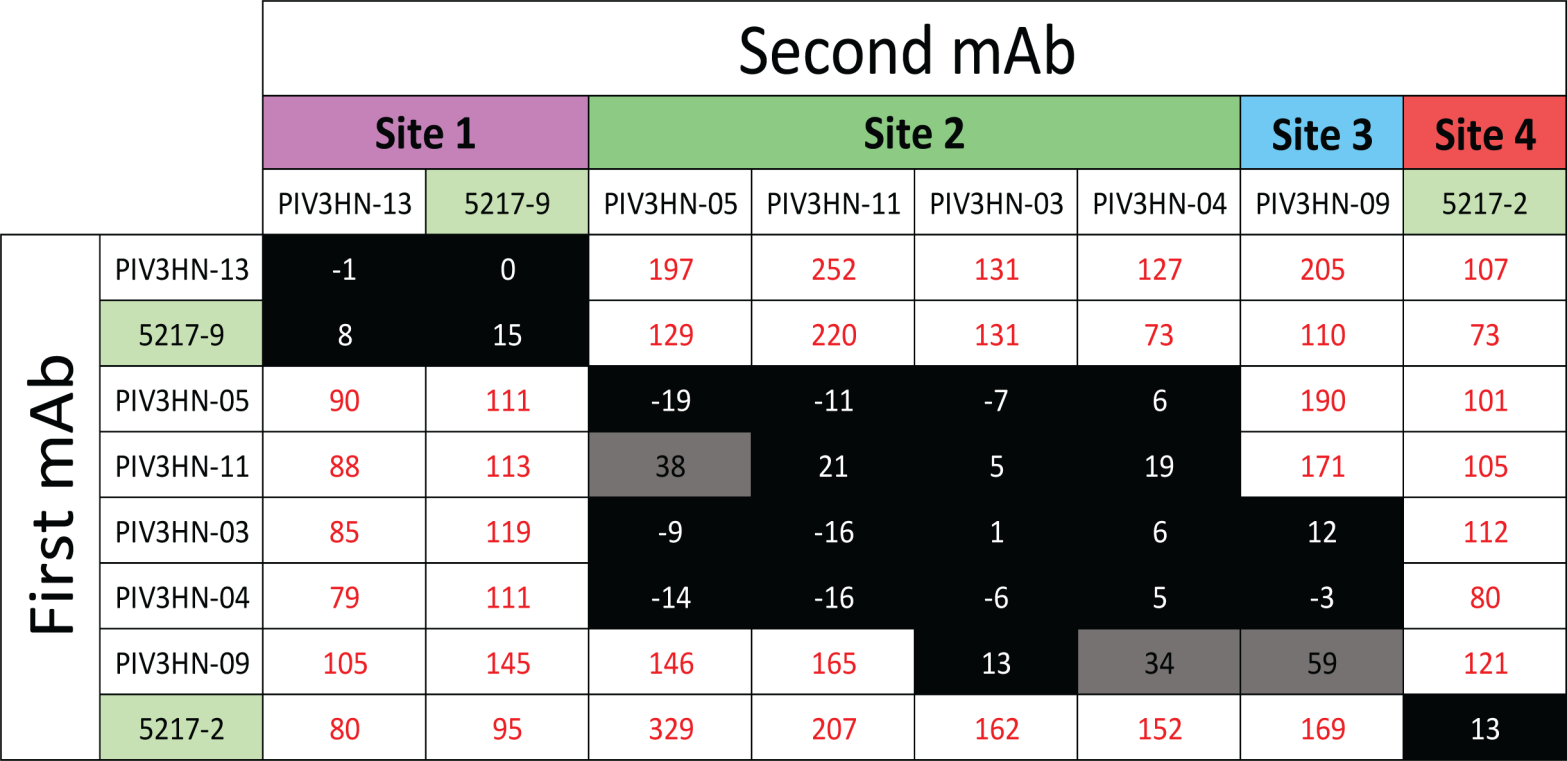
BLI epitope binning analysis of novel mAbs against previously characterized mAbs. A heat map showing the results from a binning experiment with six of the previously characterized mAbs (PIV3HN-13, PIV3HN-05, PIV3HN-11, PIV3HN-03, PIV3HN-04, PIV3HN-09) and the two new mAbs (5217-2 and 5217-9). Black indicates complete competition (≤33%), gray indicates partial competition (34%–66%), and white indicates no competition (≥67%). The formula to calculate the competition percentage is as follows: %Competition = (signal from first mAb with second mAb/signal from first mAb) × 100%. The addition of mAb 5217-2 led to the discovery of a new epitope on PIV3 HN, designated as antigenic site 4, while mAb 5217-9 bound to the previously characterized antigenic site 1. Both newly identified mAbs from this study are highlighted in green.

To further investigate the antigenic binding profiles of the newly characterized mAbs alongside those previously identified, epitope mapping was performed via competitive biolayer interferometry (BLI), a technique that measures interactions by analyzing interference patterns (51). By examining changes in the interference pattern, we were able to determine whether antibodies competed for the same binding region when introduced sequentially. The goal was to determine whether the newly isolated mAbs recognized unique epitopes or shared binding sites with the previously described mAbs. The epitope binning experiment was performed in a pairwise combinatorial format, in which each mAb was tested against itself and every other antibody in the panel. Previously, three epitope groups on the PIV3 HN protein, termed antigenic site 1, site 2, and site 3, were mapped using this method (47). The addition of the newly generated mAbs into the binning assay led to the discovery of a new binding site designated as antigenic site 4. Notably, mAb 5217-2 demonstrated exclusive binding to this new epitope, as evidenced by its unique binding profile and lack of competition with the antigenic site 1–3 antibodies. This suggests that the PIV3 HN protein possesses an additional accessible binding site that has not been defined. In contrast, mAb 5217-9 was found to compete with mAbs classified within antigenic site 1, such as PIV3HN-13 (Fig. 3). Together, these binning results expand our understanding of the antigenic landscape of the PIV3 HN protein.

### Site 3 mAb PIV3HN-09 binds opposite to site 1 mAb PIV3HN-13

**Fig 4:**
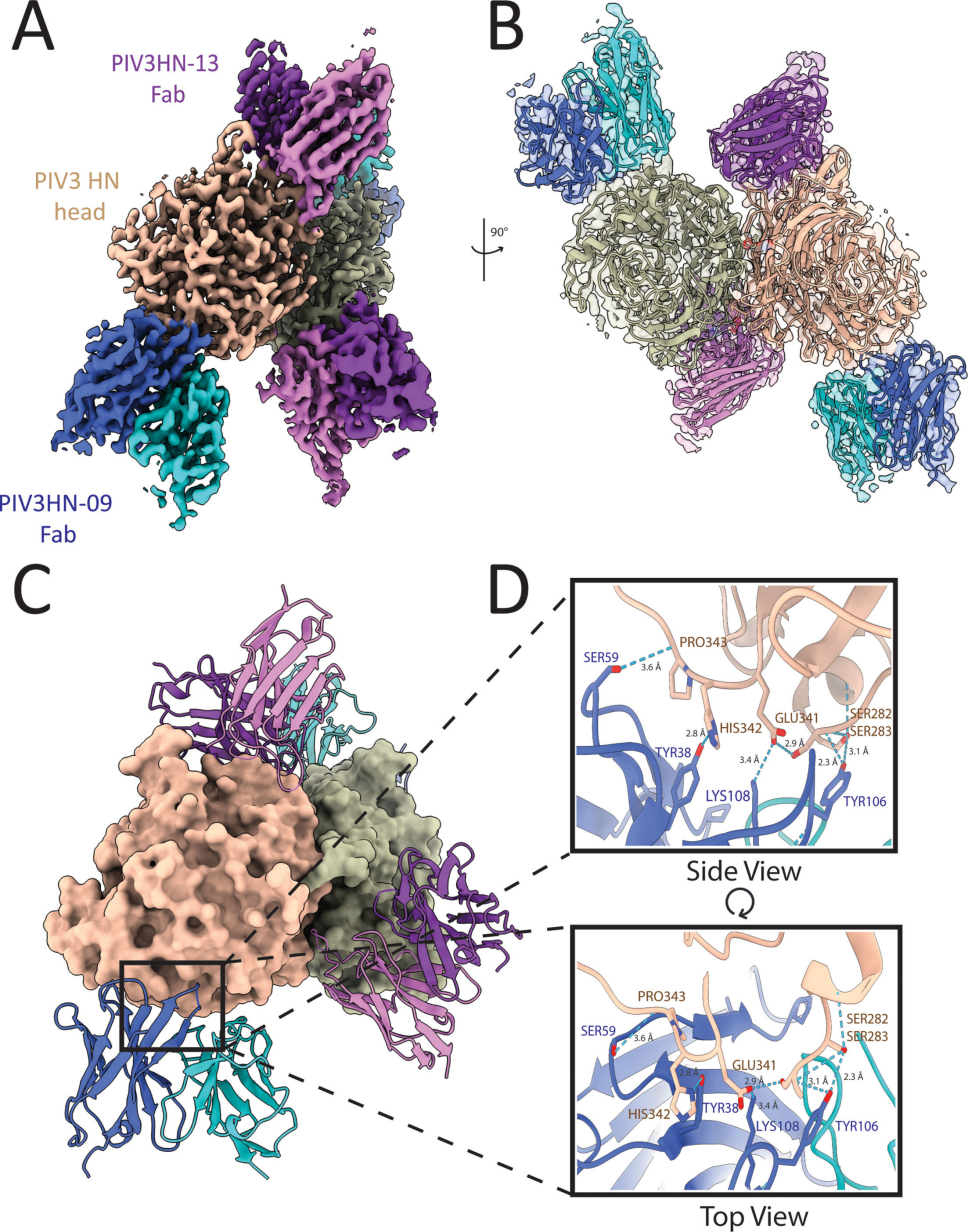
Cryo-EM structure of antigenic site 3 mAb PIV3HN-09 binding to the PIV3 HN head. (**A**) Cryo-EM density map of a PIV3 HN dimer (tan and olive green) bound to PIV3HN-09 Fabs (blue) and PIV3HN-13 Fabs (purple). (**B**) Alternative view of the atomic model (ribbon representation) fitted into the cryo-EM density (transparent surface), demonstrating a good model-to-map fit. (**C**) Surface representation of the HN proteins with bound Fab fragments, PIV3HN-09 heavy chains are colored dark blue and light chains are light blue. (**D**) Detailed view of the interaction interface between the HN protein and the PIV3HN-09 heavy chain, highlighting key interactions. Hydrogen bonds are depicted as dotted lines, with distances labeled in angstroms (Å). The top panel shows a side view of the binding interface, while the bottom panel provides a top-down perspective.

While the structures of mAbs targeting antigenic sites 1 and 2 have been previously elucidated, we sought to further define the neutralizing epitopes on the PIV3 HN protein, including antigenic site 3 and the newly discovered antigenic site 4. The antigenic site 3-directed mAb, PIV3HN-09, was previously identified as a neutralizing mAb capable of inhibiting viral fusion and reducing PIV3 lung viral titers in a hamster infection model (47). We determined the structure of mAb PIV3HN-09 in complex with the PIV3 HN protein using cryo-EM. The Fab of PIV3HN-09 was complexed with the PIV3 HN protein (along with the previously discovered antigenic site 1 mAb PIV3HN-13) and visualized using single-particle cryo-EM, allowing us to resolve the binding interface at high resolution. The final electron density map was resolved at a resolution of 2.95 Å obtained from 198,000 particles, and a predicted model from AlphaFold3 was fit into the map (Fig. 4A and B). Manual model building was performed using Coot, in which amino acid side chains and backbone conformations were adjusted to fit the cryo-EM density map. After model adjustment, refinement and validation were carried out in Phenix, which included evaluation of stereochemistry and Ramachandran outliers (Table S1 and Fig. S2). Structural analysis reveals that mAb PIV3HN-09 binds near the HN dimer interface at the opposite end of the antigenic site 1 mAb, PIV3HN-13 (Fig. 4A through C). Closer examination of the binding interface shows that the epitope is primarily recognized through interactions mediated by the heavy chain of the antibody. All three complementarity-determining region (CDR) loops of the heavy chain contribute to binding by engaging multiple PIV3 HN surface residues. Specifically, the HCDR1 residue Tyr38 forms a hydrogen bond with His342 of the PIV3 HN protein at a distance of 2.8 Å. Ser59 at the HCDR2 interacts with Pro343 (3.6 Å) of the PIV3 HN protein via a hydrogen bond. Tyr106 on the HCDR3 forms separate hydrogen bonds with Ser282 and Ser283 (2.3 Å and 3.1 Å, respectively). A final HCDR3 residue Lys108 forms a hydrogen bond with Glu341 on HN (3.4 Å) (Fig. 4C and D). Notably, the light chain of mAb PIV3HN-09 does not appear to contribute to antigen binding, as no significant contacts are observed between light chain residues and the HN protein.

### Site 4 mAb 5217-2 binds opposite to the F-HN binding interface

**Fig 5:**
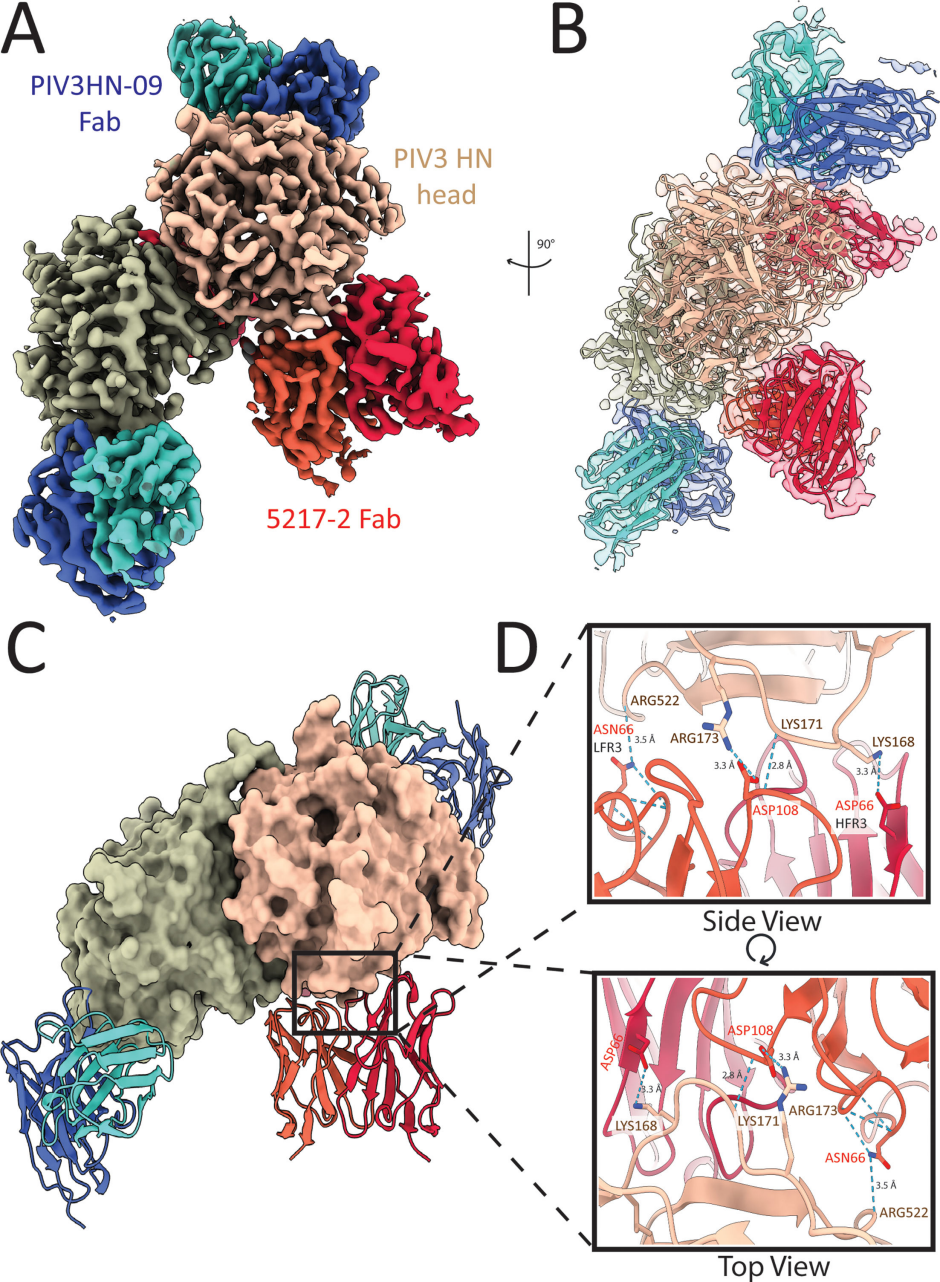
Cryo-EM structure of antigenic site 4 mAb 5217-2 binding to the PIV3 HN head. (**A**) Cryo-EM density map of a PIV3 HN dimer (tan and olive green) bound to 5217-2 Fabs (red) and PIV3HN-09 Fabs (blue). (**B**) Alternative view of the atomic model (ribbon representation) fitted into the cryo-EM density (transparent surface), demonstrating a good model-to-map fit. (**C**) Surface representation of the HN proteins with bound Fab fragments, 5217-2 heavy chains are colored dark red and light chains are orange. (**D**) Detailed view of the interaction interface between the HN protein and the 5217-2 heavy and light chains, highlighting key contact residues. Hydrogen bonds are depicted as dotted lines, with distances labeled in angstroms (Å). The top panel shows a side view of the binding interface, while the bottom panel provides a top-down perspective.

To complete the structural characterization of the four known antigenic sites on the PIV3 HN surface, we utilized cryo-EM to resolve the antigenic site 4 epitope interactions of mAb 5217-2. In this case, we leveraged the now-known epitope of mAb PIV3HN-09 by complexing this mAb and mAb 5217-2 with the PIV3 HN protein. We obtained a 3.29 Å resolution electron density map from 238,000 particles (Fig. 5A and Fig. S3 and Table S1). Structural analysis revealed that the mAb 5217-2 binds to the apex of the PIV3 HN head domain, positioned distal to the F-HN binding interface (Fig. 5A and B). Two heavy chain contacts predominantly drive the interaction, with an additional contribution from a single light chain residue. Unlike the PIV3HN-09 epitope, which engages multiple CDR loops, binding of the 5217-2 Fab relies largely on a single CDR loop. HCDR3 residue Asp108 forms two stabilizing hydrogen bonds with Arg173 (3.3 Å) and Lys171 (2.8 Å). Additional support for the interaction comes from framework residues: HFR3 residue Asp66 forms a hydrogen bond with Lys168 (3.3 Å), and LFR3 residue Asn66 forms a hydrogen bond with Arg522 (3.5 Å) (Fig. 5D).

### Molecular basis of cross-protection from PIV1 and PIV3 by the site 2 mAb PIV3HN-05

**Fig 6:**
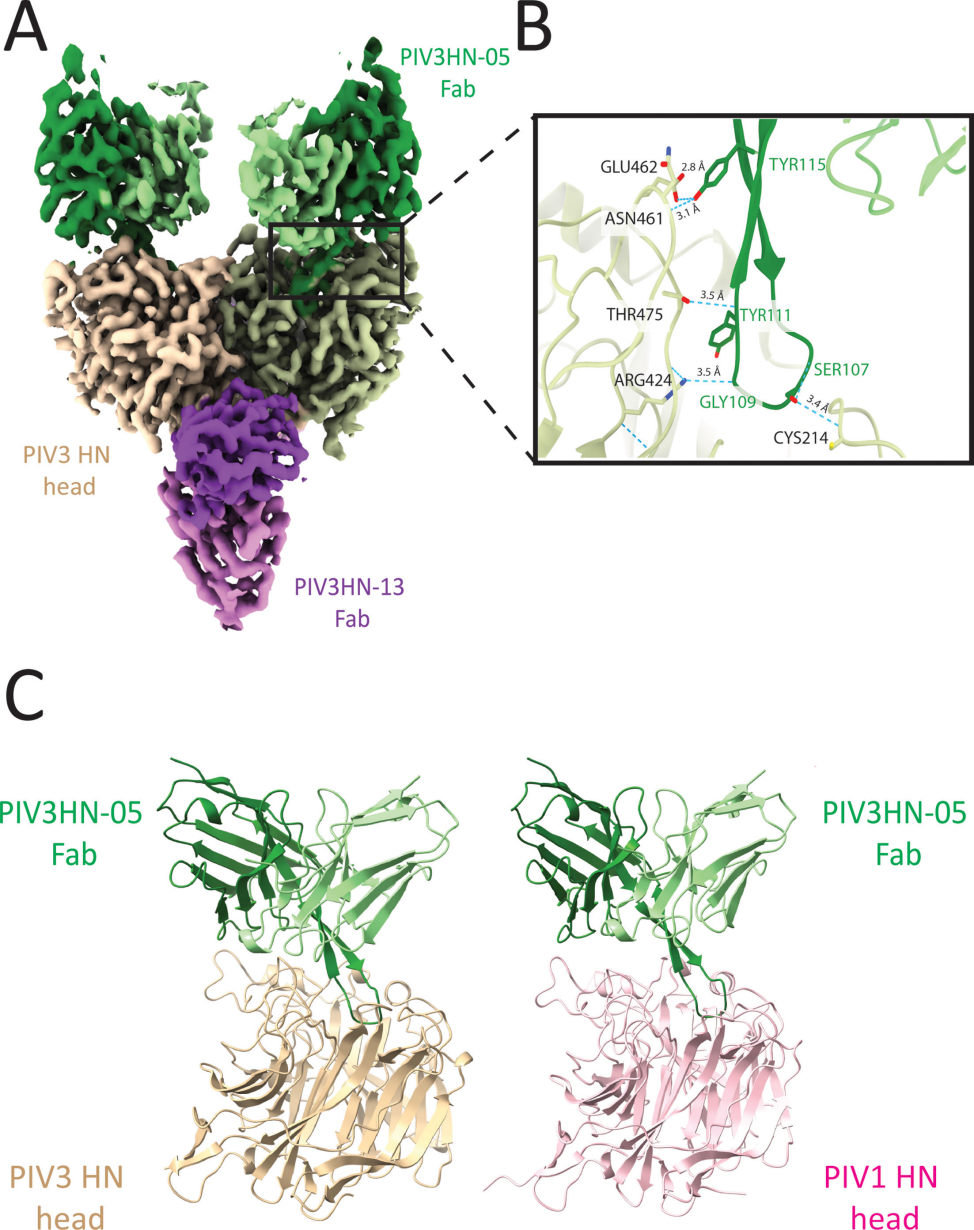
Updated cryo-EM structure of antigenic site 2 mAb PIV3HN-05 binding to the PIV3 HN head and evaluation of cross-reactivity in a PIV1 HN model. (**A**) Cryo-EM density map of a PIV3 HN dimer (tan and olive green) bound to PIV3HN-05 Fabs (green) and PIV3HN-13 Fabs (purple). (**B**) Detailed view of the interaction interface between the HN protein and the HCDR3 loop of PIV3HN-05 Fab. The Fab does not directly interact with the HN sialic acid active site (residues 234–323) but inserts into the active site, occupying the space. Hydrogen bonds are depicted as dotted lines, with distances labeled in angstroms (Å). (**C**) The bottom panel shows the overall structure of the PIV3 HN head domain in complex with the PIV3HN-05 Fab. To evaluate the potential mechanism behind PIV1 cross-reactivity, the PIV1 HN ectodomain was structurally aligned with the PIV3 HN head, and the binding site of the PIV3HN-05 Fab was compared between the two.

The potent neutralizing activity of antigenic site 2 mAbs is attributed to their targeting of the sialic acid binding site on the PIV3 HN protein, where they likely interfere with receptor binding and/or prevent viral release by blocking the interaction of the PIV3 HN protein with sialic acid (47). Notably, the antigenic site 2 mAb, PIV3HN-05, has demonstrated both prophylactic and therapeutic efficacy against PIV3 and exhibited cross-neutralization activity against PIV1, with an IC_50_ of 180 ng/mL (47). To better understand the molecular basis for this cross-neutralization, we revisited the structural characterization of the mAb PIV3HN-05 epitope. The initial cryo-EM map had disordered density within the variable region, which limited the resolution and the elucidation of the key Fab-HN interactions. Using the updated CryoSPARC package, we were able to re-pick a larger number of particles and pool them with previously selected particles using improved templates. This enabled more effective 2D and 3D classification to remove resolution-limiting particles. It was also followed by a “rebalance orientation” job to lower the orientation dominancy of the preferred views. Applying the latest version of non-uniform refinement in CryoSPARC using a tighter manual mask ultimately allowed us to generate a higher-resolution cryo-EM map of the PIV3 HN protein in complex with PIV3HN-05 Fab and PIV3HN-13 Fab at 3.29 Å resolution from 343,000 particles, providing a clearer understanding of the mode of binding (Fig. 6A, Fig. S4, and Table S1).

The structure revealed that the interaction between PIV3HN-05 Fab and the PIV3 HN protein is mediated exclusively by the heavy-chain complementarity-determining region 3 (HCDR3) loop. This loop fits deeply within the binding pocket of the PIV3 HN sialic acid binding site, likely limiting the ability of the protein to bind to the host cell sialic acid-containing receptors. This interaction is mediated primarily by five hydrogen bonds between the HCDR3 of the Fab and HN active site. Residue Tyr115 forms two stabilizing hydrogen bonds with Glu462 (2.8 Å) and Asn461 (3.1 Å), Tyr111 interacts with Thr475 (3.5 Å), Ser107 interacts with Cys214 (3.4 Å), and Gly109 interacts with Arg424 (3.5 Å) (Fig. 6B). These contacts suggest a mechanism in which the Fab physically blocks the sialic acid binding site, therefore inhibiting viral attachment and entry into the host cell. Since we previously showed PIV3HN-05 cross-neutralized PIV1, we overlaid the HCDR3 loop onto PIV1 HN to assess the mechanism behind the cross-reactivity for PIV1 (Fig. 6C) (47). The HCDR3 loop appears to insert into the sialic acid binding pocket of PIV1 HN. This observation suggests a potential shared mechanism of neutralization against both PIV1 and PIV3, although this remains to be experimentally confirmed.

Additionally, we aligned the HN head domains of PIV3 and PIV1 and compared residues within the PIV3HN-05 epitope (Fig. S5). Several contact residues were fully conserved between PIV3 and PIV1, while the remaining substitutions retained similar chemical and structural properties. The primary differences involved variations in amino acid side chain length, suggesting that the overall epitope landscape is largely preserved between the two viruses.

**Fig 7:**
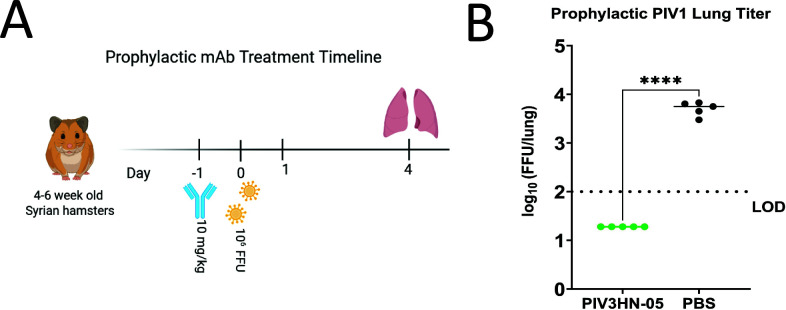
*In vivo* evaluation of PIV3HN-05 against PIV1 challenge. (**A**) Experimental timeline of the hamster study. Syrian hamsters (*n* = 10) received a single dose of PIV3HN-05 (10 mg/kg) 1 day prior to the PIV1 viral challenge. Lungs were harvested 4 days post-challenge, and viral titers were assessed via plaque assay. (**B**) Results of the plaque assay show a reduction in lung viral load in the PIV3HN-05 treated group compared to the PBS control. Statistical significance was determined using a two-tailed unpaired *t*-test, revealing a significant difference between the groups (****P < 0.0001). Animals with undetectable viral titers were plotted as the limit of detection (LOD) divided by 2.

To further evaluate the protective efficacy of the PIV3HN-05 mAb against PIV1, we conducted an *in vivo* prophylactic challenge study. For this study, we utilized Syrian golden hamsters as the animal model, as they support PIV1 replication in both the lung and nasopharynx. Hamsters received an intraperitoneal injection of mAb PIV3HN-05 at a dose of 10 mg/kg or phosphate-buffered saline (PBS) as a control, administered 24 h before the intranasal PIV1 challenge. Four days after the challenge, animal lungs were harvested and homogenized for viral titration via plaque assay (Fig. 7A). Compared to the PBS-treated group, which exhibited an average of 6.8 × 10^3^ ffu/mL per lung, the PIV3HN-05 mAb-treated group showed complete inhibition of viral replication, with no detectable plaques at the limit of detection, indicating effective reduction of PIV1 in the lungs (Fig. 7B). These findings indicate that mAb PIV3HN-05 provides protection not only against PIV3 but also against PIV1, likely due to its ability to block HN binding to host cell receptors through a conserved mechanism of neutralization.

### Site 1–4 mAbs bound to the PIV3 HN head

**Fig 8:**
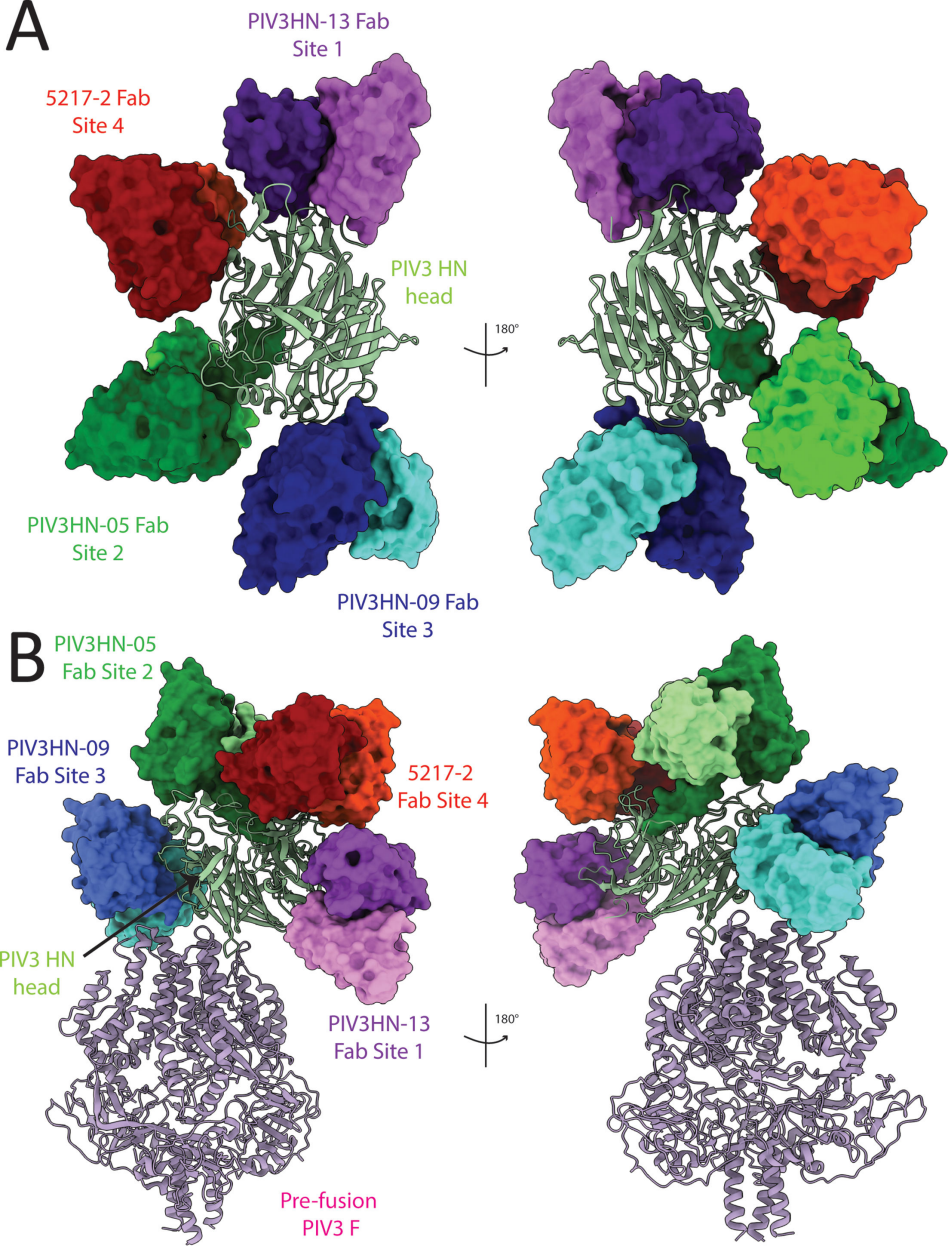
Structural mapping of antigenic sites 1–4 Fabs bound to the PIV3 HN head domain in complex with the PIV3 F protein. (**A**) A model showing the PIV3 HN head domain simultaneously bound by the four antigenic site-specific Fabs: antigenic site 1 PIV3HN-13, antigenic site 2 PIV3HN-05, antigenic site 3 PIV3HN-09, and antigenic site 4 5217-2. This model provides a comprehensive representation of the epitope location on the HN head, illustrating the relative position and accessibility of each site. (**B**) A model of the PIV3 HN head domain with antigenic sites 1–4 Fabs bound in the context of the PIV3 F protein, generated using a structure of the HN dimer (PDB 1V2I) (19), prefusion F (PDB 6MJZ) (43), and the HN-F interaction map (EMB 27550) (52). This model shows the orientation and relative positioning of each Fab when the HN is engaged with the F protein.

To gain further insight into the potential mechanism of neutralization, we fit the structures of the PIV3 HN-specific mAbs onto the PIV3 F-HN complex cryo-ET map (EMD-27550), which captured the interactions of the PIV3 F and HN proteins on authentic viral particles (Fig. 8A and B) (52). This structural alignment revealed that mAb PIV3HN-09 binds adjacent to the F-HN interaction site. The location of this binding site is consistent with previous functional data showing that mAb PIV3HN-09 has fusion-inhibiting activity (47). Comparison of the antigenic site 4 binding position relative to the F-HN interface reveals that it is situated adjacent to antigenic site 2 (Fig. 8A and B). Antigenic site 2-specific mAbs bind within the sialic acid-binding pocket of the PIV3 HN protein, whereas structural alignment demonstrated that the antigenic site 4 mAb binds just outside this pocket, adjacent to the receptor-binding region. Despite not directly overlapping the sialic acid binding site, the antigenic site 4 antibody 5217-2 exhibited potent neutralizing activity.

To evaluate the relevance of these epitopes in circulating strains, we analyzed sequences from clinical isolates and compared mAb binding residues (Fig. S6) (22). Doing this revealed that the majority of the residues within the defined mAb epitopes are conserved across strains, supporting the potential clinical breadth of the mAbs.

### Site 4 mAb 5217-2 prevents viral replication in Syrian hamster lungs

**Fig 9:**
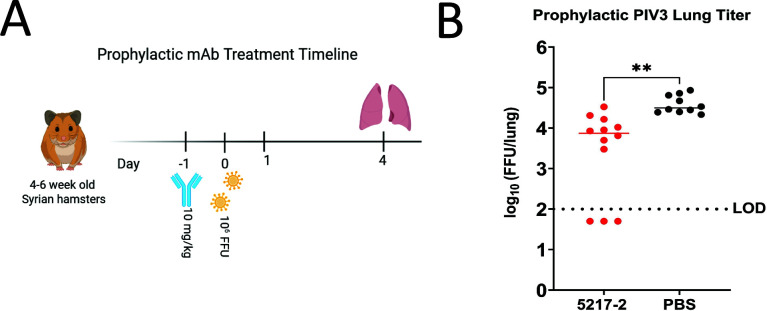
*In vivo* evaluation of 5217-2 against PIV3 challenge. (**A**) Experimental timeline of the hamster study. Syrian golden hamsters were treated prophylactically with 5217-2 (10 mg/kg) or PBS 24 h prior to intranasal PIV3 challenge. Lungs were harvested 4 days post-challenge, and viral titers were assessed via plaque assay. (**B**) Results represent pooled data from two independent experiments. Two animals were excluded from the analysis due to failed viral infection evidenced by a plaque count of less than 10 in the 1/10 dilution well. Plaque assay analysis demonstrated a 1-log reduction in lung viral titers in the 5217-2 treated group compared to PBS controls. Statistical significance was determined using a two-tailed unpaired *t*-test, revealing a significant difference (**P = 0.0032) between the groups. Animals with undetectable viral titers were plotted as the LOD divided by 2.

Given the proximity of mAb 5217-2 to the receptor-binding region on the PIV3 HN protein, together with its potent *in vitro* neutralization and complement deposition activity, we next evaluated whether this mAb could confer protection *in vivo* in a prophylactic setting. Syrian golden hamsters were treated 24 h before infection with 10 mg/kg of mAb or PBS control via intraperitoneal injection. Hamsters were then challenged with PIV3 intranasally, lung samples were collected, and viral load in the lungs was quantified via plaque assay (Fig. 9A). Administration of mAb 5217-2 resulted in a significant reduction in viral burden compared to the control-treated animals. PBS-treated hamsters exhibited a mean viral load of 2.2 × 10^4^ ffu/lung, whereas animals treated with mAb 5217-2 showed significantly lower titers, averaging 4.7 × 10^3^ ffu/lung (Fig. 9B). This represents approximately a 4.5-fold reduction in viral replication within the lung.

## DISCUSSION

With no approved vaccines or antiviral therapies currently available for PIVs, there is a critical need for the development of effective preventative and therapeutic strategies. Neutralizing mAbs have shown clinical success against several respiratory viruses demonstrating their potential to mitigate disease severity in vulnerable populations. However, to date, no mAbs have been approved for the treatment or prevention of PIV infections, highlighting a significant gap in available interventions against this virus. In this study, we introduced two mAbs that target the PIV3 HN antigen and characterized the binding, viral neutralization ability, *in vivo* prophylactic activity, and structural properties of these mAbs. With this, we were able to determine additional epitopes on the PIV3 HN protein and demonstrated its potential as a viable target for vaccines or mAb development.

With the addition of the fourth epitope, antigenic site 2 of PIV3 HN remains the primary target of the most potent neutralizing mAbs. Although the antigenic site 2 mAb PIV3HN-03 was previously reported as the strongest inducer of complement deposition among the panel, we observed that the antigenic site 4 mAb 5217-2 exhibited increased complement deposition relative to mAb PIV3HN-03 (47). Complement deposition did not directly correlate with neutralization activity, suggesting that epitope-dependent structural features influence Fc-mediated effector function independently of viral neutralization.

In addition to the efficacy studies, we determined the structures of mAbs targeting antigenic sites 3 and 4 on the PIV3 HN protein to gain insight into the mechanism of protection. Structural analysis revealed that the antigenic site 3-specific mAb, PIV3HN-09, binds adjacent to the interface between the PIV3 HN and F proteins. Given that viral entry depends on coordinated HN-F interactions to trigger F protein-mediated membrane fusion (21, 23), the position of PIV3HN-09 suggests that it may interfere with this activation step; however, this hypothesis has not been directly tested. Consistent with its ability to inhibit viral fusion (47), the orientation of PIV3HN-09 at the HN-F interface suggests that its binding imposes steric constraints that limit efficient HN-mediated activation of the F protein.

Structural reanalysis of the PIV3 HN protein-mAb PIV3HN-05 interaction reveals that the HCDR3 loop directly occupies the sialic acid binding pocket of the HN protein, potentially blocking the HN’s ability to bind to sialic acid on the host cell. Consistent with this binding mode, mAb PIV3HN-05 exhibits potent neutralizing activity and cross-reactivity with PIV1, underscoring the sialic acid binding pocket as a key functional target for HN-directed neutralization. The cross-reactive properties of mAb PIV3HN-05 are further supported by the high degree of structural and sequential conservation between the HN proteins of PIV1 and PIV3.

In contrast, the structure of mAb 5217-2 revealed that the antigenic site 4 epitope is positioned at the apex of the HN head domain, adjacent to the receptor-binding region targeted by mAb PIV3HN-05. Although the antigenic site 4 epitope does not directly overlap this active site, its proximity to this functional region suggests that mAb binding at antigenic site 4 could influence HN function through sterically limiting receptor access or by restricting the conformational flexibility of the HN head domain required for receptor binding. This proposed mechanism is supported by the observed reduction in viral load *in vivo* following prophylactic administration of mAb 5217-2. Together, these data suggest that epitopes adjacent to the receptor-binding site can mediate protective immunity, highlighting antigenic site 4 as a functionally relevant region of PIV3 HN.

Several limitations of this study should be considered. First, the *in vivo* efficacy was evaluated exclusively in a prophylactic setting, prior to the establishment of PIV infection. As a result, it remains unclear whether these mAbs would be effective in a therapeutic context, which is an important factor in clinical applications. In addition, the *in vivo* assessment was limited to measuring viral burden and we did not evaluate clinical signs of disease or histopathology. As a result, we cannot determine whether differences in complement activation influence disease severity beyond the effect of viral replication. Second, although murine models are often an effective method of measuring antibody-mediated protection, species-specific differences in complement regulation between rodents and humans may influence Fc effector functions and limit the translational interpretation of these mAbs (53). Finally, Fc effector function was primarily tested through complement-induced cytotoxicity; however, mAbs can also engage additional Fc-dependent mechanisms, such as recruiting innate immune cells to induce phagocytosis or NK-mediated cytotoxicity. Together, these limitations highlight the need for future studies incorporating therapeutic challenge models and a more comprehensive evaluation of Fc-mediated effector mechanisms to further define the protective efficacy of HN-targeting antibodies.

In summary, our study advances understanding of the antigenic landscape of the PIV3 HN protein, highlighting the distinct roles of various epitopes in mediating antibody protection. The identification of multiple antigenic sites on PIV3 HN with distinct neutralization and complement-inducing profiles provides a framework for rational vaccine design strategies that elicit antibodies targeting conserved and critical regions, such as antigenic site 2. By integrating epitope binning, plaque reduction neutralization assays, structural characterizations, and *in vivo* efficacy studies, we concluded that mAbs targeting antigenic sites 2 and 4 are the most promising targets for further investigation. Our findings emphasize the robust protective capacity of antigenic site 2-targeting antibodies, particularly mAb PIV3HN-05, which binds a critical and conserved region on the PIV3 HN protein. To further understand the basis for its cross-protective activity, future studies should investigate the structural interactions between mAb PIV3HN-05 and the HN protein of PIV1. Additionally, further studies should be done to evaluate the *in vivo* efficacy and structural interactions of other antigenic site 2 mAbs, such as PIV3HN-11, which was shown to inhibit fusion of PIV3 to host cells (47). Together, these efforts will help guide the development of broadly protective monoclonal antibodies targeting the HN protein of PIV3, as well as those from other parainfluenza viruses.

## MATERIALS AND METHODS

### Cell lines and viruses

Freestyle293-F cells (ThermoFisher, Cat: R79007) were cultured in Freestyle 293 media (ThermoFisher, Cat: 12338026). ExpiCHO-S cells (ThermoFisher, Cat: A29127) were cultured in ExpiCHO Expression Medium (ThermoFisher, Cat: A2910001). LLC-MK2 cells (ATCC, CCL-7) were cultured in Opti-MEM I + GlutaMAX (ThermoFisher, Cat: 51985034) supplemented with 2% fetal bovine serum. PIV1 (Cat: NR-48680) and PIV3 (Cat: NR-3233) were obtained from BEI resources. The virus was diluted into virus medium (Opti-MEM, 1:500 trypsin-EDTA, 1:100 CaCl_2_, and 1% antibiotic-antimycotic) and added to LLC-MK2 cells at an MOI of 0.01. Virus was propagated at 5% CO_2_ for 3–5 days until CPE was observed. The virus was subsequently harvested using a sucrose freeze/thaw method. The virus-containing sucrose solution was aliquoted, flash-frozen in liquid nitrogen, and stored at −80°C until further use.

### Production of PIV3 HN viral protein

A plasmid construct for the head domain of PIV3 HN was designed based on the wild-type PIV3 strain C243 HN sequence (GenBank JN089924.1) with the N-terminal cytoplasmic tail and transmembrane domains removed and a hexahistidine tag added. Plasmid containing the HN head domain insert was transformed into DH5a *Escherichia coli* competent cells prior to plasmid isolation and transfection. The recombinant protein was expressed in Freestyle293-F cells. Plasmids were diluted in Opti-MEM medium at a final concentration of 1 µg/mL. The transfection reagent PEI MAX (Kyfora Bio, Cat: 24765) was added at a ratio based on the culture volume and incubated with the plasmid mixture for 30 min before adding to the cells. Cultures were centrifuged at 10,000 RPM for 15 min, and then the supernatant was passed through a 0.45 µm filter. Protein was purified through a HisTrap Excel pre-packed column (Cytiva, Cat: 17371206) per the manufacturer’s instructions. Purified protein was buffer exchanged into PBS and stored at −80°C until use.

### Peripheral blood mononuclear cells isolation

Leukocyte filters obtained from Shepeard Blood Bank (Augusta, GA) were emptied into sterile tubes and flushed twice with PBS. The collected blood was carefully layered on top of pre-warmed Lymphocyte Separation Medium (Corning, Cat: 25-072-CV) and then centrifuged at 2,500 RPM for 30 min using minimal brake and acceleration (set at 1) to not disturb the layers. The plasma was removed, and the buffy coat was carefully extracted and added to Dulbecco’s Modified Eagle Medium (DMEM) (Corning, Cat: 10-013-CV). The PBMCs were washed twice with DMEM (300 × *g* for 15 min), and PBMCs were aliquoted into cryovials with Stemcell Media A (Stemcell Technologies, Cat: 03801), and 10% DMSO (Thermo Scientific, Cat: J66650.AE) was added to minimize cell damage during freezing. Cryovials were placed in a cryogenic freezing container and stored at –80°C overnight. For long-time preservation, samples were transferred to liquid nitrogen storage.

### 5217-2 and 5217-9 mAb discovery

PBMCs from blood of human donors were blocked with Trustain FcX (Biolegend, Cat: 422302) for 30 min, washed once in PBS, and subsequently stained according to the following protocol: 5 µL CD19-APC (Biolegend, Cat: 302212), 1.25 µL IgM-FITC (Southern Biotech, Cat: 2020-02), 1.25 µL IgD-FITC (Southern Biotech, Cat: 2030-02), 2.5 µL GhostRed710 (Cytek Biosciences, Cat: 13-0871-T100), and 5 µL of PIV3 HN conjugated to PE (Biolegend, Cat: 405204). The cells were stained for 30 min, washed twice in PBS, and immediately sorted on a Moflo Astrios EQ (Beckman Coulter). Prior to antigen-specific sorting, nine pneumococcal proteins (PcpA, PspA, PsaA, PhtE, Ply, NanA, PcsB, StkP, PiuA) and two viral proteins, PIV HN and RSV B2F Pre, were biotinylated using the EZ-Link NHS-PEG4 Biotinylation kit as per the manufacturer’s protocol (ThermoFisher, Cat: 21455). Streptavidin-conjugated PE (Invitrogen, Cat #S866) was slowly added to the biotinylated antigens at a fluorophore to protein molar ratio of 4:1, adding 1 uL of streptavidin-PE every 20 min for a total of five additions, and stored on ice away from light. PBMCs were suspended in FACS buffer (PBS, 2% FBS) (Gibco, Cat # A5256701), 2% goat serum (Gibco, Cat #16210072), 0.5 M EDTA, and Fc-blocked with Human TruStain FcX (BioLegend, Cat #422301) on ice for 30 min. The sorted cells were processed through 10× sequencing (Pleasanton, CA) at the UGA Genomics and Bioinformatics Core, according to the manufacturer’s protocol (10× Genomics, Protocol: CG000736). After 10× processing and analysis, paired heavy and light chain variable sequences were obtained (Table S2) and cloned by Twist Biosciences (San Francisco, CA) into their respective cloning vector (heavy: pTwist CMV hIgG1, lambda: pTwist CMV hIgL2, kappa: pTwist CMV hIgK).

### Plasmid transformation into *E. coli* competent cells

Plasmids from 10× sequences were ordered from Twist Biosciences and transformed into *Escherichia coli* DH5α competent cells (Invitrogen, Cat: 18258012). The cells were incubated with plasmid DNA (1 µL) and heat shocked before incubating in LB broth medium for 1 h and plated on ampicillin agar plates overnight. Isolated colonies were then added to a 5 mL culture of LB broth with ampicillin (1:1,000) and then expanded to a 200 mL culture 8 h later to shake overnight. The DNA plasmids were extracted using the E.Z.N.A Plasmid DNA Maxi Kit (Omega Bio-Tek, Cat: D6922-04) according to the manufacturer’s instructions. The resulting plasmid DNA was sterile filtered for later use in transfections.

### Plasmid transfection into Freestyle 293-F cells and ExpiCHO cells

Both Freestyle 293F cells and ExpiCHO-S cells were used to transfect the PIV mAbs. *In vitro* studies utilized mAb produced from ExpiHO-S cells, while the *in vivo* studies used mAb produced from Freestyle 293F cells. For Freestyle 293F transfections, heavy and light chain plasmids were diluted in Opti-MEM medium at a final concentration of 1 µg/mL. The transfection reagent PEI MAX (Kyfora Bio, Cat: 24765) was added at a ratio based on the culture volume and incubated with the plasmid mixture for 30 min before adding to the cells. Transfections using ExpiCHO-S cells (ThermoFisher, Cat: A29127) were performed according to the ExpiFectamine CHO transfection kit (ThermoFisher, Cat: A29129) based on the Max Titer Protocol.

### Purification of mAbs

mAbs were purified 7 days post-transfection in Freestyle 293F cells or 14 days post-transfection in ExpiCHO-S cells. Cultures were centrifuged at 10,000 RPM for 15 min and then the supernatant was passed through a 0.45 µm filter. A HiTrap Protein G column (Cytiva, Cat: 17040503) was used to purify the supernatant according to the manufacturer’s instructions. The eluted mAbs were spun in 30 kDa MWCO concentrators (Millipore, Cat: UFC903096) and buffer exchanged with PBS. The concentration was measured on a NanoDrop spectrophotometer using the IgG setting.

### Enzyme-linked immunosorbent assay

To measure binding to the PIV3 HN protein, 384-well plates were coated with 2 µg/mL of antigen overnight at 4°C. The plates were blocked for 1 h with 2% non-fat dry milk supplemented with 2% goat serum. Plates were then washed three times with water, and primary mAbs were applied to wells for 1 h at 37°C. Following another three washes with water, a secondary antibody, goat anti-human IgG Fc-AP (Southern BioTech, Cat: 2048-04) at a dilution of 1:4,000 in 1% blocking solution was added. After incubating for an hour, the plates were washed five times with PBS-T (0.05% Tween-20 in PBS), and substrate solution (1 mg/mL PNPP disodium salt hexahydrate, ThermoFisher) was added to each well. The plates were incubated in the dark for 1 h at room temp before reading the optical density at 405 nm on a Biotek plate reader.

### Complement deposition cytotoxicity assay

Biotinylated PIV3 HN antigen was coupled with FluoSpheres Neutravidin beads (Invitrogen, Cat: F8775) at a 1:1 ratio of antigen (µg): beads (µL) for 2 h at 37°C. The beads were washed with 5% PBS-BSA and resuspended 1:100 in 0.1% PBS-BSA. The mAbs were diluted to 2.5 µg/mL in a 96 U-bottom plate and incubated for 2 h with 10 µL of antigen-specific beads. A corresponding isotype negative control mAb, along with a no-mAb control, was used alongside the sample mAbs. The bead-mAb complexes were then washed with 0.1% PBS-BSA. Guinea pig complement (MP Biomedicals, Cat: 64283) was diluted 1:50 in R-10 buffer (RPMI-1640 + 10% FBS). The diluted complement was then incubated with the bead-mAb complex for 15 min and then washed twice with PBS. Fluorescein-conjugated goat anti-guinea pig complement C3 (MP Biomedicals, Cat: 55385) was diluted 1:100 in PBS and incubated for 15 min in the dark. Complexes were washed twice with PBS and resuspended in a final volume of 150 µL PBS. Samples were read on the Cytek Aurora.

### Flow cytometry with PIV3-infected cells

Biotinylated mAbs were incubated with streptavidin-conjugated PE (BioLegend, Cat: 405204) on ice for 1 h and stored at 4°C for later use. LLC-MK2 cells were cultured in a T225 flask at 37°C, 5% CO_2_ to 80%–90% confluency prior to infection. Cells were washed with PBS twice and infected with PIV3 at an MOI of 0.01. The virus was incubated with the cells for 1 h at 37°C, 5% CO_2_, with gentle rocking every 10 min. Virus culture media (OptiMEM + 5 µg/mL trypsin-EDTA + 100 µg/mL CaCl_2_ + 1% 100× anti-anti) was added to the flask and incubated for 48 h. After incubation, the cells were digested using Versene (Gibco, Cat: 15040-066) at 37°C, 5% CO_2_ for 45 min. Cells were washed twice with FACS buffer and incubated in FACS for 30 min on ice. Aliquots of 1 million cells were incubated with their respective PE-conjugated mAbs at 2.5 µg/mL for 30 min on ice in the dark. Cells were again washed twice with FACS buffer and fixed for 15 min with 4% paraformaldehyde. Finally, the cells were resuspended in 1 mL of FACS buffer. Samples were read on the Cytek Aurora.

### PIV3 mAb plaque reduction neutralization assay

LLC-MK2 cells were seeded in 24-well flat-bottom plates and incubated at 37°C, 5% CO_2_ for 2 days to reach confluency. Following the incubation period, 5217-2 and 5217-9 mAbs were serially diluted and incubated at a 1:1 ratio with PIV3 for 1 h at room temperature. The media was removed from the 24-well plates, and 50 µL/well of the virus/mAb mixture was added on top of the cells and rocked at 37°C, 5% CO_2_ for 1 h. Cells were then overlaid with 0.75% methylcellulose dissolved in Opti-MEM with 5 µg/mL trypsin-EDTA and 100 µg/mL CaCl_2_ and incubated for 4 days. After incubation, the cells were fixed with 10% neutral buffered formalin for 45 min and washed once with water. The plaques were immuno-stained by first blocking the plates for 1 h with a block comprised of 2% non-fat milk and 2% goat serum in PBS-T. The plates were washed two times with water and the primary mAb, PIA174, was then added at a dilution of 1 µg/mL in the block and incubated for 1 h at room temp. After washing two times with water, HRP-conjugated goat anti-human IgG (Southern BioTech, Cat: 2048-05) diluted 1:2,000 in the block was added for an hour. The plates were washed three times with water and TrueBlue peroxidase substrate (Seracare, Cat: 5510-0030) was added to each well and rocked for no longer than 10 min and washed once with water. The plates were allowed to dry overnight and were manually counted using a stereoscope. IC_50_ values were calculated using GraphPad Prism10.

### Epitope binning

Biolayer interferometry on a Gator Prime instrument was performed for epitope binning. Anti-His (HIS) Probes (GatorBio, PN:160009) were equilibrated in kinetics buffer. A baseline measurement was recorded by dipping the biosensors into wells containing the kinetics buffer for 60 s. Next, biosensors were loaded with PIV3 HN at a concentration of 100 µg/mL for 150 s, followed by another baseline measurement. The loaded biosensors were then dipped into wells containing either mAb PIV3HN-01, PIV3HN-03, PIV3HN-04, PIV3HN-05, PIV3HN-09, PIV3HN-11, PIV3HN-13, 5217-2, or 5217-9 at a concentration of 100 ug/mL for 300 s. The biosensors were then dipped into another set of the same mAbs listed previously for 300 s to test for a second association. Each antibody was tested for competition against itself and all other antibodies, with assays performed in both directions by using each antibody in both the first and second association steps.

### Expression and purification of Fabs

Fab fragments PIV3HN-01, PIV3HN-09, PIV3HN-13, and 5217-2 were generated by incubating the full-length mAbs with immobilized papain overnight at 37°C, following the Pierce Fab Preparation Kit (ThermoFisher, Cat: 44985). The Fc portion was removed using HiTrap MabSelect columns (Cytiva, Cat: 28408253) according to the manufacturer’s instructions.

### Production of antigen + Fab complexes and cryo-EM grid preparation

Fab fragments (0.7 mg/mL) were combined with PIV3HN (0.35 mg/mL) at a twofold molar excess and incubated overnight at 4°C. The complex was purified on a Superdex 200 10/300 SEC column (Cytiva, Cat: 28990944) with 50 mM Tris-HCl pH 7.5 and 150 mM NaCl. The purified complex peak was confirmed using SDS-PAGE gel stained with Coomassie blue and concentrated to 1 mg/mL for further use. A FEI Vitrobot Mark IV was used to prepare samples onto Quantifoil R 1.2/1.3 300 copper mesh grids (Ted Pella, Cat: 658-300-CU). The grids were glow-discharged before adding 5 μL of sample and fast incubated before being blotted and plunged into liquid ethane.

### Cryo-EM data collection and processing

After screening the prepared grids, 4,000 movies (PIV09 complex) and 6,300 movies (5217-2 complex) were collected on Titan-Krios 300 KV microscope equipped with Gatan-K3 camera (Table S1 for details). Movies were then imported into the CryoSPARC package (54), where patch motion correction and patch CTF estimation were performed. Blob picker was used to pick particles, which were then purified using multiple 2D classification cycles. Selected particles were then used to make multiple *ab initio* 3D reconstructions which were used for further 3D classification (heterogeneous refinement in CryoSPARC) to obtain the final particle set. Final 3D reconstruction was performed using the non-uniform refinement job to obtain a 2.95 Å (PIV09 complex) and a 3.29 Å (5217-2 complex) resolution map. We used the sharpened map from this refinement for model building and model refinement, and the sharpened map from DeepEMhancer for visualizations and figures.

### Model building and validation

PIV3 HN dimer model and Fab aa sequences were all imported into AlphaFold3 (55). After fitting and fixing the backbone and sidechains with precision into the map using Coot (56), PHENIX (57) was used to do a 3D real-space refinement and validation (Table S1). Output models were used to fit into the cryo-EM map using ChimeraX (58).

### Structural fitting into cryo-ET density

A model of the PIV3 HN dimer (PDB 1V2I) (19) and a PIV3 F prefusion-stabilized trimer (PDB 6MJZ) (43) were fit into the cryo-ET density map of the PIV3 HN-F assembly (EMD-27550) (52) using ChimeraX (58). After aligning each component into the corresponding density manually, the “fit in map” tool was used to further validate the fit. After establishing this HN-F arrangement as a reference, the mAbs-HN complex was overlaid onto the fitted HN dimer through the HN domain to preserve the same orientation within the map.

### Prophylactic/therapeutic treatment and viral challenge of Syrian hamsters

Four- to six-week-old female Golden Syrian hamsters (Charles River Laboratories #049) were individually housed at Florida State Universities’ animal facility. One day before the viral challenge, groups were prophylactically treated with their respective mAb (10 mg/kg) through intraperitoneal (IP) injection. After 24 h, hamsters were anesthetized with isoflurane for 2 min before intranasally (IN) infecting with PIV3 (10^6^ PFU/mL). Hamsters were humanely euthanized using pentobarbital injection 4 days post-infection. Lungs were collected and washed with PBS and added to 2 mL of cold Opti-MEM and homogenized for viral titration. If not used immediately, lung samples were stored at −80°C before homogenization.

### Syrian hamster lungs viral titration

After homogenization, the lung homogenate was serially diluted in cold Opti-MEM media. The diluted homogenate was added to an LLC-MK2 cell monolayer in a 24-well tissue culture plate (200 µL/well) and rocked for 1 h at 37°C, 5% CO_2_. The following steps of the titration assay were performed as described earlier.

## Data Availability

Raw data for each relevant figure are available at 10.6084/m9.figshare.31460275. The cryo-EM structures of PIV3HN-09, 5217-2, and PIV3HN-05 generated by this study are available at the Protein Data Bank with the following accession codes: 9YQ6, 9YTS, and 9ZB5. The corresponding map for each of these complexes has been deposited in the Electron Microscopy Data Bank and can be found using the following accession codes: EMD-73325, EMD-73493, and EMD-73975.

## SUPPLEMENTAL MATERIAL

Supplemental material jvi.00502-26-s0001.pdf Tables S1 and S2; Fig. S1 to S6.

ASM does not own the copyrights to Supplemental Material that may be linked to, or accessed through, an article. The authors have granted ASM a non-exclusive, world-wide license to publish the Supplemental Material files. Please contact the corresponding author directly for reuse.
