# Supplementary material for: Molecular basis for protection and cross-protection by human antibodies targeting the parainfluenza virus hemagglutinin-neuraminidase protein
Source: J Virol. 2026 Jul 31;100(8):e00502-26. doi: 10.1128/jvi.00502-26 (PMC13483390; doi:10.1128/jvi.00502-26)
Supplement: Supplementary file 1 [file jvi.00502-26-s0001.pdf]

Table S1. Cryo-EM data collection, refinement, validation, and model-building statistics.

	PIV3HN + PIV3HN-09 + 5217-2	PIV3HN + PIV3HN-09 + PIV3HN-13	PIV3HN + PIV3HN-13 + PIV3HN-05
**Data collection and processing**			
Scope	Titan Krios	Titan Krios	Titan Krios
Magnification	105,000	59,000	59,000
Voltage (kV)	300	300	300
Electron exposure (e–/Å^2)	60	60	60
Defocus range (μm)	0.8-2.8	0.8-2.8	0.8-2.8
Camera	Gatan K3	DE-Apollo	DE-Apollo
Pixel size (Å)	0.88	0.79	0.79
Symmetry imposed	C2	C2	C2
Initial particle images (no.)	3,800,000	150,000	1,200,000
Final particle images (no.)	238,000	22,000	287,000
Map resolution (Å)<br>FSC threshold	3.29<br>0.143	6.57<br>0.143	2.88<br>0.143
Map resolution range (Å)	2-6	5.5-10	2.3-7
**Refinement**			
Initial model used (PDB code)	Alphafold3	Alphafold3	7UR4/Alphafold
Model resolution (Å)<br>FSC threshold	2.61<br>0.143	2.94<br>0.143	3.29<br>0.143
Model resolution range (Å)	2.8-6	2.4-6	2.5-8
Map sharpening *B* factor (Å^2)	116.1	84.0	135.1
R.m.s. deviations<br>Bond lengths (Å)<br>Bond angles (°)	 0.003<br>0.564	 0.003<br>0.588	 0.003<br>0.717
Validation<br>Clash-score<br>Poor rotamers (%)	 6.47<br>4.07	 9.97<br>2.50	 7.01<br>4.63
Ramachandran plot<br>Favored (%)<br>Allowed (%)<br>Disallowed (%)	 90.99<br>8.56<br>0.45	 95.04<br>4.96<br>0.00	 86.82<br>12.61<br>0.56
PDB accession code	9YTS	9YQ6	9ZB5
EMDB accession code	EMD-73493	EMD-73325	EMD-73975
EMPIAR accession code	TBD	TBD	TBD

**Table S2. Nucleotide sequences of parainfluenza virus monoclonal antibodies.**	
**5217-2 HC**	gaggtgcacctcctggagtctgggggaggcctggtccagcctggggggtcccgcagaatctcctgtgcagcctctggattctcttttggcagctatgccatgagctgggtccgccaggctccagggaaggggctggagtgggtctcgactataactagtagtggtgaaagcacagactacgcagactccgtgaagggccggttcatcatctccagagacaacgccaagaacacgctggatctcctaatgaacagcctgagagccggggacacggccgtttactactgtgcgaagggggggggcagtagtactgagcgctatggacgtctggggccaagggactacggtcaccgtctcctca
**5217-2 KC**	gacatccagttgacccagtctccatcttccctgtctgcatctgtaggagacagggtcaccatcacttgccaggcgagtcacgacattagtaactatctaaattggtatcagcagaaaccagggaaagcccctaagctcctgatctacgatgcatccaatttggaaacaggggtcccaccaaggttcagtggaggtggatctgggacacattttagtctcaccatcagcagcctgcagcctgaagacattgcaacatattactgtcaacaggatgatagtctcccattaactttcggccctgggaccaaggtggaaatcaaa
**5217-9 HC**	gaggtgcagctgttggagtcgggggaggcttggtacagcctggggggtccctgagactctcctgtgcagcctctggattcacctttagcagccatgccatgagctgggtccgccaggctccagggaaggggctggagtgggtctcaactattggtagtagtggcattagtacatactacacagactccgtgaagggccgcttcaccatctccagagacaattccaagaacacgctgtttcttcaactgaacagcctgagagccgaggacacggccgtctattactgtgaagtgggggggatcaaatcgcgtcttcctactggggccagggaaccctggtcaccgtctcctca
**5217-9 LC**	gacatccagatgacccagtctccttccaccctgtctgcatctgttggggatagagtcaccatcacttgccgggccagtgagaatattaatagttggttggcctggtatcagcagaaaccagggaaagcccctaaactcctgatctacaaggcgtctagtttacaaagtggggtcccatcaaggttcagcggcagtagatctgggacagaattcactctcaccatcagtagcctgcagcctgatgattttgcaacttattactgccaacagtataatacttatttctcgtacacttttggccaggggaccaagttggagatcaaa

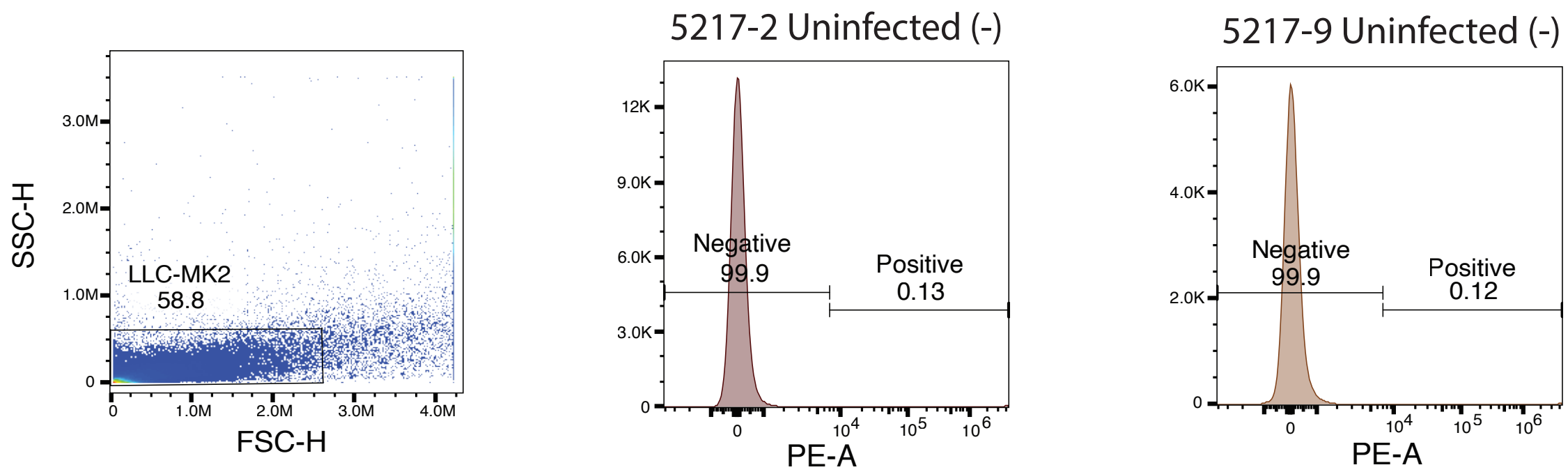

Figure S1. Flow cytometry analysis of mAb binding to uninfected LLC-MK2 cells. Cells incubated with each mAb in the absence of virus showed minimal staining, demonstrating that mAb binding is specific to viral antigen and not to the cells themselves.

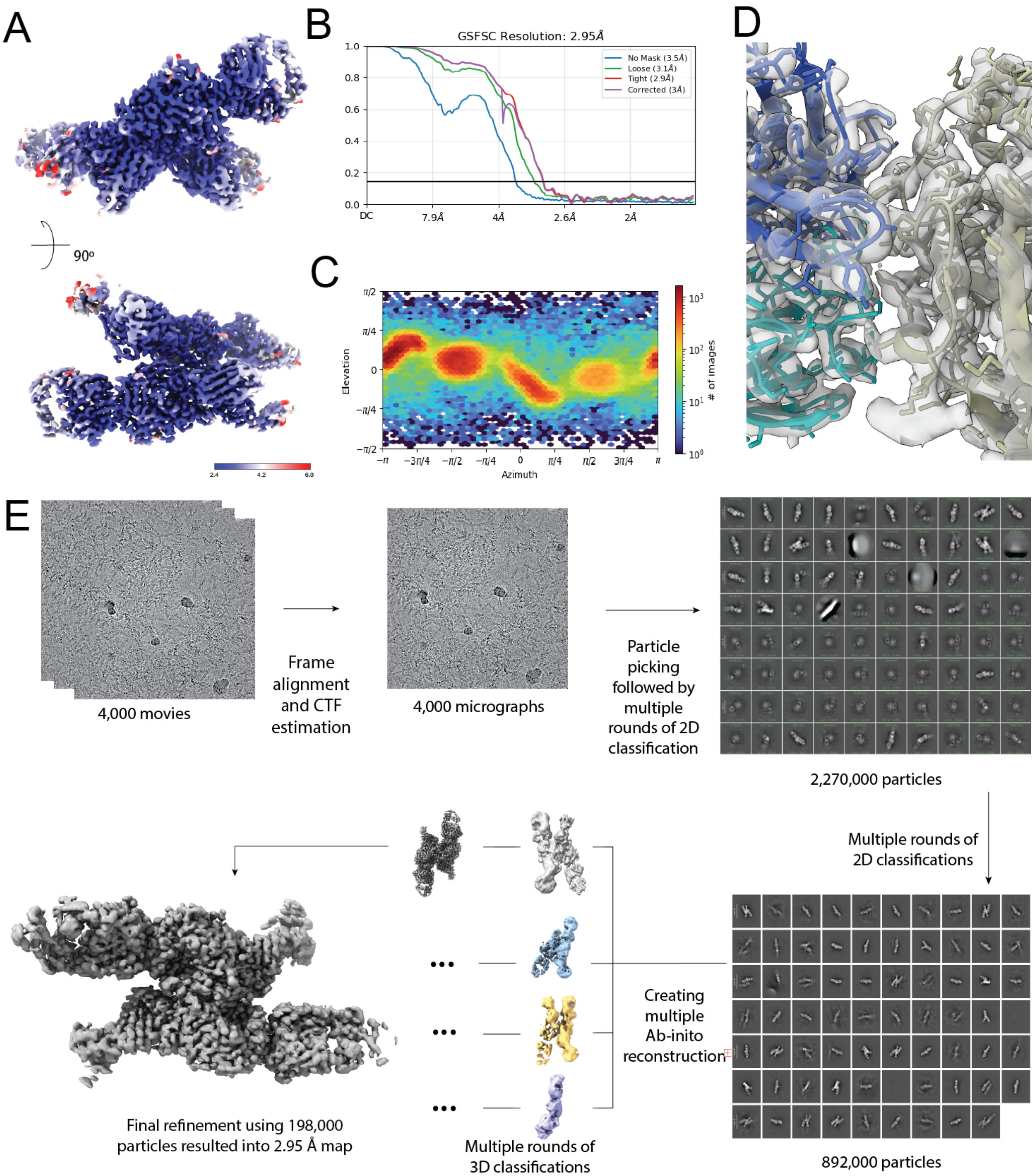

Figure S2. PIV3HN-09 cryo-EM processing workflow. (A) Local resolution map obtained for PIV3 HN protein bound to the PIV3HN-09 Fab and PIV3HN-13 Fab. (B) GSFSC curve of the refined cryo-EM map. (C) Particle distribution map for the final refinement. (D) Model to map fit example of the PIV3 HN protein interface with PIV3HN-09 Fab. (E) Overall cryo-EM data processing workflow.

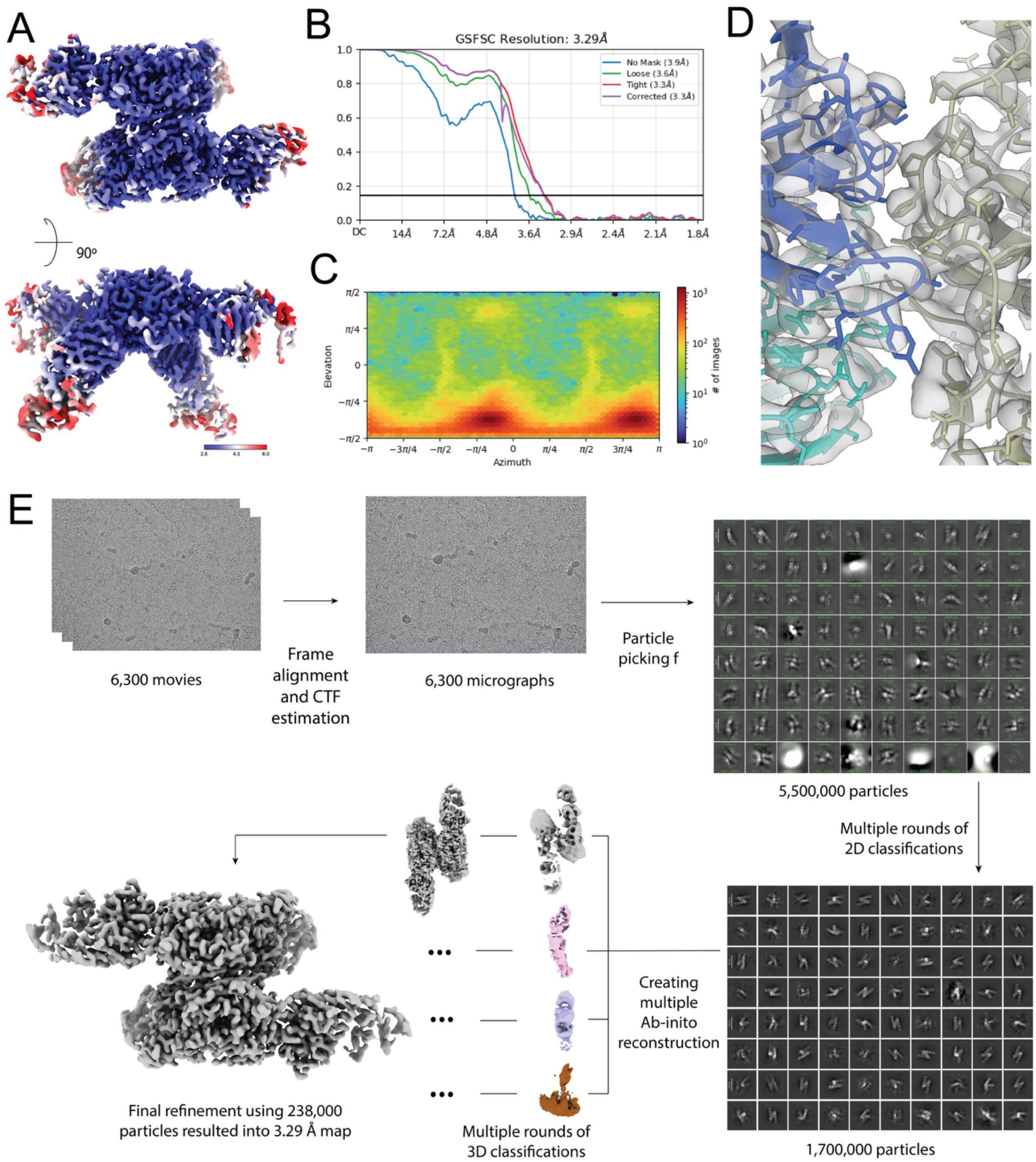

Figure S3. 5217-2 cryo-EM processing workflow. (A) Local resolution map obtained for PIV3 HN protein bound to 5217-2 and PIV3HN-09 Fabs. (B) GSFSC curve of the refined cryo-EM map. (C) Particle distribution map for the final refinement. (D) Model to map fit example of the PIV3 HN protein interface with 5217-2 Fab. (E) Overall cryo-EM data processing workflow.

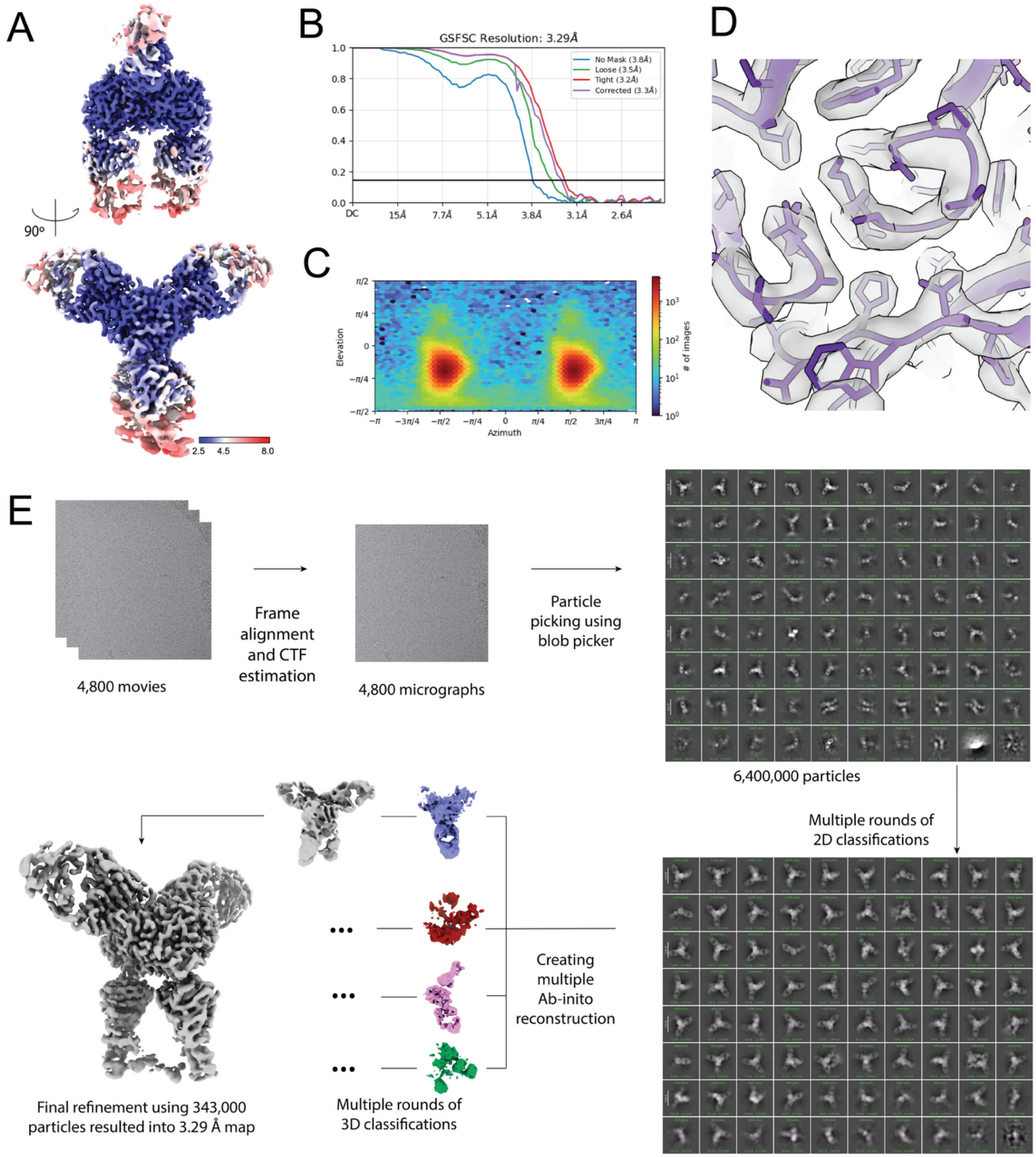

Figure S4. PIV3HN-05 cryo-EM processing workflow. (A) Local resolution map obtained for PIV3 HN protein bound to PIV3HN-05 and PIV3HN-13 Fabs. (B) GSFSC curve of the refined cryo-EM map. (C) Particle distribution map for the final refinement. (D) Model to map fit example of the PIV3 HN protein interface with PIV3HN-05 Fab. (E) Overall cryo-EM data processing workflow.

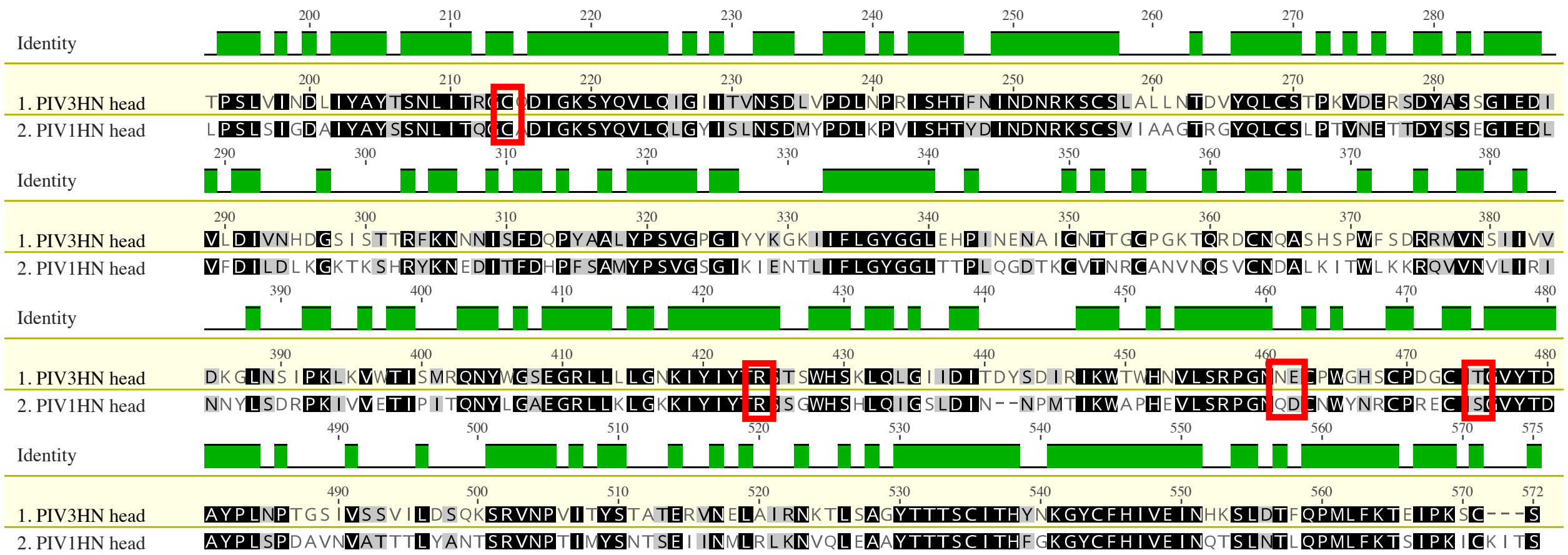

Figure S5. Sequence alignment of the PIV3 HN head and PIV1 HN head domains. Red boxes indicate the PIV3 HN residues contacted by PIV3HN-05 and are shown for comparison with the corresponding residues in PIV1 HN. Several residues are conserved, while the remaining substitutions are structurally and chemically similar. The primary differences among the non-conserved amino acids are variation in amino acid side chain lengths.

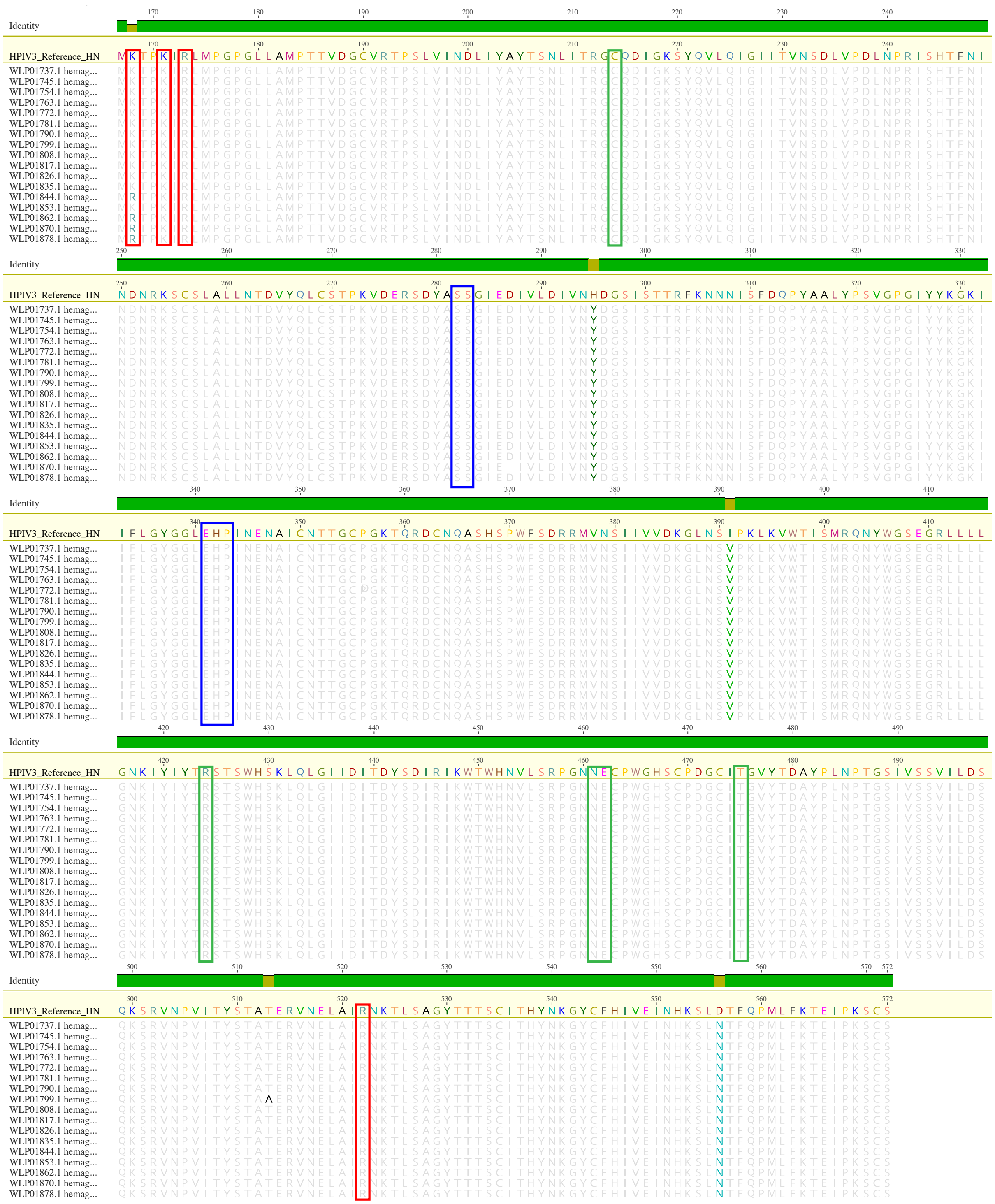

Figure S6. Sequence conservation of mAb binding sites in clinical isolates. Multiple sequence alignment of PIV3 HN from clinical isolates highlighting the residues of the mapped mAb epitopes. The blue boxes indicate PIV3HN-09 mAb binding, the red boxes indicate 5217-2 mAb binding, and the green boxes indicate PIV3HN-05 binding. These data suggest that the majority of epitope residues are maintained across circulating strains.
